# Evaluation of the respiratory route as a viable portal of entry for *Salmonella* in poultry via intratracheal challenge of *Salmonella* Enteritidis and *Salmonella* Typhimurium^1^

**DOI:** 10.3382/ps.2013-03602

**Published:** 2014-01-21

**Authors:** G. Kallapura, M. J. Morgan, N. R. Pumford, L. R. Bielke, A. D. Wolfenden, O. B. Faulkner, J. D. Latorre, A. Menconi, X. Hernandez-Velasco, V. A. Kuttappan, B. M. Hargis, G. Tellez

**Affiliations:** * Department of Poultry Science, University of Arkansas, Fayetteville 72701; † Facultad de Medicina Veterinaria y Zootecnia, Universidad Nacional Autonoma de Mexico, 04510, Mexico

**Keywords:** broiler, intratracheal challenge, *Salmonella* Enteritidis, *Salmonella* Typhimurium

## Abstract

Experimental and epidemiological evidence suggests that primary infection of *Salmonella* is by the oral-fecal route for poultry. However, the airborne transmission of *Salmonella* and similar enteric zoonotic pathogens has been historically neglected. Increasing evidence of *Salmonella* bioaerosol generation in production facilities and studies suggesting the vulnerabilities of the avian respiratory architecture together have indicated the possibility of the respiratory system being a potential portal of entry for *Salmonella* in poultry. Presently, we evaluated this hypothesis through intratracheal (IT) administration of *Salmonella* Enteritidis and *Salmonella* Typhimurium, as separate challenges, in a total of 4 independent trials, followed by enumeration of cfu recovery in ceca-cecal tonsils and recovery incidence in liver and spleen. In all trials, both *Salmonella* Enteritidis and *Salmonella* Typhimurium, challenged IT colonized cecae to a similar or greater extent than oral administration at identical challenge levels. In most trials, chickens cultured for cfu enumeration from IT-challenged chicks at same dose as orally challenged, resulted in an increase of 1.5 log higher *Salmonella* Enteritidis from ceca-cecal tonsils and a much lower dose IT of *Salmonella* Enteritidis could colonize ceca to the same extent than a higher oral challenge. This trend of increased cecal colonization due to IT challenge was observed with all trails involving week-old birds (experiment 2 and 3), which are widely considered to be more difficult to infect via the oral route. Liver-spleen incidence data showed 33% of liver and spleen samples to be positive for *Salmonella* Enteritidis administered IT (10^6^ cfu/chick), compared with 0% when administered orally (experiment 2, trial 1). Collectively, these data suggest that the respiratory tract may be a largely overlooked portal of entry for *Salmonella* infections in chickens.

## INTRODUCTION

There is good experimental and epidemiological evidence that primary infection of *Salmonella* is by the oral-fecal route, along with an established infectious dose (Blaser and Newman, 1982; White et al., 1997; Galanis et al., 2006). Although the common route of transmission for many zoonotic pathogens such as *Salmonella* is direct ingestion, the inhalation of infectious particles should also be considered (López et al., 2012). Overall, the potential airborne transmission of zoonotic enteric pathogens such as *Salmonella* has been largely neglected, even though several studies have suggested that airborne transmission of *Salmonella* is possible (Wathes et al., 1988; Harbaugh et al., 2006; Oliveira et al., 2006).

*Salmonella* bioaerosol generation, fate, and transport at various stages of commercial poultry production have been studied. Further, emphasis is laid on knowledge of survivability of *Salmonella* in these bioaerosols, as a means to assess the transport and subsequent risk of exposure and infection of poultry (Douwes et al., 2003; Jarquin et al., 2009; Brooks et al., 2010; Dungan, 2010; Cox and Pavic, 2010; Fallschissel et al., 2010). Reports have demonstrated that fan-driven spread of *Salmonella* within the hatching cabinet and hatchery incubator is possible (Berrang et al., 1995).

Modern poultry production with densely stocked and enclosed production buildings is often accompanied by movement of large volumes of air through the house, by negative pressure, to improve ventilation and environmental conditions for broiler growth, and can carry pathogens such as *Salmonella* in the form of bioaerosols, contaminated dust, or both (Davies and Wray, 1994; Baykov and Stoyanov, 1999; Byrd et al., 1999; Gast et al., 2004; Heber et al., 2006; Bull et al., 2006; Chinivasagam et al., 2009).

*Salmonella*-contaminated or -infected live poultry entering the processing plant are the primary source of processing-plant contamination, which may affect the microbial quality of the end product (McCapes et al., 1991; Clouser et al., 1995; Mitchell et al., 2002). In this regard, Harbaugh et al. (2006) hypothesized that rapid airborne *Salmonella* infection (less than 4 h) occurs in turkeys and before slaughter, suggesting the prevalence of airborne *Salmonella* in commercial poultry (Harbaugh et al., 2006). They predicted multiple points of increased exposure before slaughter may occur when birds are taken off feed for 3 to 6 h before loading. This can increase the contact of the flock with *Salmonella* through increased ingestion of feces or through increased aerosolization due to scratching of the litter in the barn in search of food (Ramirez et al., 1997; Corrier et al., 1999). Thus, increased *Salmonella*-laden dust in the air may increase the risk of infection due to inhalation of *Salmonella* bioaerosols. Similarly, large amounts of dust generated during the load-out process and improper cleaning and disinfection of crates are also likely to contaminate chickens (Corrier et al., 1999). Finally, once turkeys arrive at the slaughter plant, they are kept in cooling sheds until shackled. Large fans blow air on the turkeys to keep them cool during the summer months. Turkeys arriving at the cooling sheds may harbor *Salmonella* and air flow may transfer the organism to the environment and to uninfected birds. These events occur in the last few hours, and all increase exposure to *Salmonella*-laden dust (Harbaugh et al., 2006).

In this regard, our laboratory has recently hypothesized that transmission by the fecal-respiratory route may be a viable portal of entry for *Salmonella* and could explain some clinical impressions of relatively low-dose infectivity under field conditions in relation to the requisite high oral challenge dose that is typically required for infection of poultry through the oral route in laboratory studies. Presently, we compared intratracheal (**IT**) with oral (**OR**) administration of 2 pathogenic *Salmonella* serovars, *Salmonella* Enteritidis and *Salmonella* Typhimurium (**ST**), with relatively different invasiveness, on ability to colonize the ceca and liver and spleen (**LS**).

## MATERIALS AND METHODS

### Experimental Birds

Day-of-hatch, off-sex broiler chickens were obtained from Cobb-Vantress (Siloam Springs, AR) and placed in isolators with a controlled age-appropriate environment as explained further. Chickens were provided ad libitum access to water and a balanced unmedicated corn-soybean diet meeting the nutrition requirements of poultry recommended by the NRC (1994). Adequate body temperature was maintained using heat lamps within the isolators. All animal handling procedures were in compliance with Institutional Animal Care and Use Committee at the University of Arkansas. Twelve chickens for each trial were humanely killed and sampled upon arrival at the laboratory. Whole ceca-cecal tonsils (**CCT**), LS, and trachea were aseptically removed from these neonate chickens, incised, and cultured in 10 mL of tetrathionate enrichment broth (**Tet**, catalog no. 210420, Becton Dickinson, Sparks, MD) and incubated overnight at 37°C. The samples were confirmed negative for *Salmonella* by plating them on to selective brilliant green agar (**BGA**, catalog no. 70134, Sigma, St. Louis, MO) with novobiocin (**NO**, 25 µg/mL, catalog no. N-1628, Sigma).

### Salmonella *Cultures*

*Salmonella* Enteritidis and *Salmonella* Typhimurium served as challenge pathogens, both originally obtained as primary poultry isolates from the USDA National Veterinary Services Laboratory (Ames, IA). These isolates were resistant to novobiocin and were selected for resistance to naladixic acid (**NA**, 20 µg/mL, catalog no. N-4382, Sigma). For the present studies, 100 µL of *Salmonella* Enteritidis or *Salmonella* Typhimurium (depending on the trial) from a frozen aliquot was added to 10 mL of tryptic soy broth (**TSB**, catalog no. 211822, Becton Dickinson, Sparks, MD) and incubated at 37°C for 8 h. This was followed by 3 passages every 8 h into fresh TSB, for a total of 24 h, to ensure log phase growth. Postincubation, bacterial cells were washed 3 times in sterile 0.9% saline by centrifugation (1,864 × *g*, 4°C, 15 min), quantified with a spectrophotometer (Spectronic 20D+, Spectronic Instruments Thermo Scientific, Waltham, MA) at 625 nm using an established concentration curve, and diluted in sterile 0.9% saline as per required concentrations (cfu/mL) for the trials. Concentrations of *Salmonella* Enteritidis and *Salmonella* Typhimurium were also determined retrospectively by serial dilution and further plating onto BGA NO-NA for enumeration of actual cfu per milliliter used for challenge, as reported below.

### Challenge

All groups were challenged with *Salmonella* using sterile gavage needles with a 22-gauge 304 stainless-steel assembly of tubing and smooth terminal ball (1.25 mm diameter), in a volume of 0.25 mL. Oral challenge was through crop gavage. Care was taken, while challenging chickens intratracheally, to accurately insert the gavage needle into the trachea as deep as possible and then discharge a desired volume of challenge near the primary bronchi.

### Experimental Design

A set of 4 trials was carried out with 2 different *Salmonella* serovars, varying in their invasiveness, to evaluate the respiratory route as a viable portal of entry in comparison with the established oral route.

#### Experiment 1.

For this preliminary trial, 45 day-of-hatch, off-sex broiler chicks were obtained, randomly selected, divided into 3 groups (n = 15 chickens), and placed in individual isolators. Group 1 was challenged OR with a dose of 3 × 10^4^ cfu of *Salmonella* Enteritidis/chick, and groups 2 and 3 were challenged IT with 1.5 × 10^2^ or 3 × 10^4^ cfu of *Salmonella* Enteritidis/chick, respectively. A total of 0.25 mL was used for both IT and OR administration. All chickens were placed in individual isolators according to groups with unrestricted access to feed and water. Twenty-four hours postchallenge, all chickens were humanely killed and 12 chicks/group were cultured for *Salmonella* recovery in cecae. Briefly, whole CCT were aseptically removed, collected in sterile bags, homogenized, weighed, and 1:4 wt/vol dilutions were made with sterile 0.9% saline. Ten-fold dilutions of each sample, according to groups, were made in a sterile 96-well flat bottom plate and the diluted samples were plated on BGA with NO and NA, and incubated at 37°C for 24 h to enumerate total *Salmonella* cfu. Further, these saline-diluted cecal samples were enriched in an equal volume of double-strength Tet and incubated for 24 h at 37°C. Also, portions of LS and whole tracheal samples were aseptically collected in 10 mL of single-strength Tet broth for enrichment and incubated at 37°C for 24 h. Following this, all CCT, LS, and tracheal enrichment samples were plated on BGA with NO and NA plates and incubated at 37°C for 24 h to confirm the presence/absence of typical lactose-negative colonies of *Salmonella*.

#### Experiment 2.

A total of 2 trials were carried out in the second experiment with a similar experimental design as the preliminary trial. For trial 1, a total of 60 day-of-hatch, off-sex broiler chicks were obtained, randomly selected, and divided into 4 groups (n = 15 chickens). All chickens were placed in individual isolators according to groups with unrestricted access to feed and water. On d 7, all groups were challenged with a 0.25-mL suspension of *Salmonella* Enteritidis, with groups 1 and 3 challenged OR with a dose of 1 × 10^4^ and 1.5 × 10^6^ cfu of *Salmonella* Enteritidis/chick, and groups 2 and 4 challenged IT with 1 × 10^4^ and 1.5 × 10^6^ cfu of *Salmonella* Enteritidis/chick. Twenty-four hours postchallenge (d 8), all chickens were humanely killed and 12 chicks/group were cultured for only incidence of *Salmonella* for cecae and LS as described above.

For trial 2, a total of ninety day-of-hatch off-sex broiler chicks were obtained, randomly selected, and divided into 6 groups (n = 15 chickens). All chicks were placed in individual isolators according to groups, with unrestricted access to feed and water. On d 7, all groups were challenged with a 0.25-mL suspension of *Salmonella* Enteritidis, with groups 1, 3, and 5 challenged OR with a dose of 1.5 × 10^4^ and 2 × 10^6^ and 1 × 10^8^ cfu of *Salmonella* Enteritidis/chick, and groups 2, 4, and 6 were challenged IT at the same doses, respectively. Twenty-four hours postchallenge (d 8), all chickens were humanely killed and cultured for *Salmonella* recovery in cecae. Cecal cfu enumeration and incidence of *Salmonella* for cecae, LS, and tracheal samples were determined in the same manner as experiment 1.

#### Experiment 3.

For experiment 3, a relatively less invasive *Salmonella* Typhimurium served as the challenge isolate. A total of 90 day-of-hatch, off-sex broiler chicks were obtained, randomly selected, and divided into 6 groups (n = 15 chickens). On d 7, all groups were challenged with a 0.25-mL suspension of *Salmonella* Typhimurium, with groups 1, 3, and 5 challenged OR with a dose of 1.5 × 10^4^ and 2.5 × 10^6^ and 1 × 10^8^ cfu of *Salmonella* Typhimurium/chick, and groups 2, 4, and 6 were challenged IT at the same doses, respectively. Twenty-four hours postchallenge (d 8), all chickens were humanely killed and cultured for *Salmonella* recovery. Enumeration of cfu for ceca was not performed for this trial, whereas the incidence of *Salmonella* for cecae, LS, and tracheal samples was determined in the manner described above.

### Data and Statistical Analysis

Numerical data from all the trials were subjected to ANOVA (SAS Institute, 2002). Log_10_ cfu values of *Salmonella* per gram of ceca were expressed as means ± SEM and deemed significant if *P* ≤ 0.05. The data were also subjected to mean separation using Duncan's multiple range test significance. The enrichment data were expressed as positive/total chickens (%) and the percent recovery of *Salmonella* was compared using the chi-squared test of independence, testing all possible combinations to determine the significance (*P* ≤ 0.05) for these studies (Zar, 1984).

## RESULTS AND DISCUSSION

Data of experiment 1 are presented in Table 1. Neonates chicks challenged IT could colonize CCT within 24 h of challenge, indicating the respiratory route is as viable as that of the oral route under these experimental conditions. Although there were no significant differences in colonization rates in this experiment, cfu enumeration from group 3 challenged IT at same dose (3 × 10^4^ cfu/chick) as that of group 1 (OR), resulted in a numerical 1.5 log-higher recovery of *Salmonella* Enteritidis from CCT, with values 7.76 ± 0.23 and 6.25 ± 0.76, respectively, for IT and OR. Further, group 2, which was challenged with a much lower dose IT (1.2 × 10^2^ cfu/chick) of *Salmonella* Enteritidis could colonize ceca to the same (or to a numerically higher) extent than that of group 1 challenged at a higher dose OR (3 × 10^4^ cfu/chick), with values of 6.88 ± 0.41 and 6.25 ± 0.76, respectively, for IT and OR.

**Table 1.** Evaluation of intratracheal infection of chickens with *Salmonella* Enteritidis (experiment 1)^1^

Group	Dose, cfu of *Salmonella* Enteritidis/chick	Route of challenge	Log_10_ cfu of *Salmonella* Enteritidis/g of ceca content	Ceca-cecal tonsil	Liver and spleen	Trachea
1	3 × 10^4^	OR	6.2547 ± 0.7578	11/11 (100)	11/11 (100)	11/11 (100)
2	1.5 × 10^2^	IT	6.8833 ± 0.4089	12/12 (100)	12/12 (100)	12/12 (100)
3	3 × 10^4^	IT	7.7568 ± 0.2264	12/12 (100)	12/12 (100)	12/12 (100)

^1^Chicks were challenged with *Salmonella* Enteritidis on day of hatch intratracheally (IT) or orally (OR) at concentrations of 1.5 × 10^2^ or 3 × 10^4^ cfu/chick. Twenty-four hours postchallenge, ceca-cecal tonsils were cultured to enumerate log_10_ *Salmonella* Enteritidis per gram of ceca content and the data were expressed as mean ± SEM. Ceca-cecal tonsils, trachea, and liver and spleen enrichment data were expressed as positive/total chickens for each tissue sampled (% in parentheses). No significant differences (*P* < 0.05, n = 12/group) were found between the groups.

Chickens at a younger age, especially hatchlings, are very susceptible to *Salmonella* infection (Noy and Sklan, 1998; Dibner et al., 1998; Sklan and Noy, 2000; Halevy et al., 2000; Noy et al., 2001; Uni et al., 2003; Juul-Madsen et al., 2004; Yi et al., 2005; Hooshmand, 2006; Henderson et al., 2008); however, this trial shows that they can be infected via the respiratory route with around 100 cells (Table 1). Further, given at such a low dose, the cecal colonization was still equivalent to that recovered from a higher OR challenge. These results may be important, considering a report (Berrang et al., 1995) demonstrating the fan-driven spread of *Salmonella* within the hatching cabinet and hatchery incubator, which conventionally would be considered to settle on dust, fluff, and water droplets, which then ingested by these birds would lead to oral infection. Considering hatchlings will spend up to 48 h before placement in commercial brooding houses, respiratory transmission may be a possibility.

Experiment 2, trial 1 was performed to extend the evaluation with relatively older chickens, the results of which are provided in Table 2. The incidence of *Salmonella* infection was compared using 2 routes of challenge in 7-d-old chickens. At a lower dose of approximately 10^4^, the incidence of cecal colonization and positive LS were only numerically higher compared with zero positive samples in OR challenged chicks. At the higher dose of 10^6^, positive CCT samples were higher (10/12: 83.33%) for OR route of challenge compared with that of IT (6/12: 50%; Table 2).

**Table 2.** Evaluation of intratracheal infection of chickens with *Salmonella* Enteritidis (experiment 2, trial 1)^1^

Group	Dose, cfu of *Salmonella* Enteritidis/chick	Route of challenge	Ceca-cecal tonsil	Liver and spleen
1	1 × 10^4^	OR	0/12 (0)^c^	0/12 (0)^b^
2	1 × 10^4^	IT	2/12 (16.66)^bc^	1/12 (8.33)^ab^
3	1.5 × 10^6^	OR	10/12 (83.33)^a^	0/12 (0)^b^
4	1.5 × 10^6^	IT	6/12 (50)^ab^	4/12 (33.33)^a^

^a–c^Different superscripts within columns indicate significant differences (*P* < 0.05), n = 12/group.

^1^Chicks were challenged with *Salmonella* Enteritidis on d 7 intratracheally (IT) or orally (OR) at concentrations of 1 × 10^4^ or 1.5 × 10^6^ cfu/chick. Twenty-four hours postchallenge, cecal tonsils and liver and spleen were cultured in an enrichment broth. The enrichment data were expressed as positive/total chickens for each tissue sampled (% in parentheses).

Experiment 2, trial 2 was an extension of trial 1, evaluating the same objective at 3 different doses, in 1-wk-old birds, and the data are shown in Table 3. In trial 2, the ceca cfu recovery of *Salmonella* at the lowest (10^4^) and highest dose (10^8^) were similar, if not higher. However, it was interesting to note a significantly higher cecal recovery for the intermediate dose (10^6^) given IT, with values of 1.86 ± 0.40 cfu/g for OR and 3.20 ± 0.17 for IT. Overall, we observed a clear dose response curve with the IT groups compared with groups challenged OR. When incidence of recovery from cecal tonsils was compared, there was significantly greater recovery of *Salmonella* Enteritidis from the CCT following enrichment in the group receiving the lowest challenge of *Salmonella* Enteritidis (10^4^ cfu) IT compared with OR. Similarly, a significantly higher recovery of *Salmonella* Enteritidis was observed following enrichment from LS samples at the intermediate and highest challenge doses administered by IT compared with OR. As might be expected, tracheal recovery at each challenge level was significantly higher following IT challenge compared with OR. However, it is interesting to note that *Salmonella* Enteritidis was recovered from some tracheal samples following OR challenge with each of the challenge levels.

**Table 3.** Evaluation of intratracheal infection of chickens with *Salmonella* Enteritidis (experiment 2, trial 2)^1^

Group	Dose, cfu of *Salmonella* Enteritidis/chick	Route of challenge	Log_10_ cfu of *Salmonella* Enteritidis/g of ceca content	Ceca-cecal tonsil	Liver and spleen	Trachea
1	1.5 × 10^4^	OR	1.89 ± 0.38^c^	5/12 (41.66)^b^	0/12 (0)^d^	1/12 (8.33)^d^
2		IT	1.13 ± 0.40^c^	8/12 (66.66)^b^	6/12 (50)^bc^	8/12 (66.66)^bc^
3	2 × 10^6^	OR	1.86 ± 0.40^c^	8/12 (66.66)^b^	2/12 (16.66)^cd^	3/12 (25)^d^
4		IT	3.20 ± 0.17^b^	12/12 (100)^a^	10/12 (83.33)^ab^	11/12 (91.66)^ab^
5	1 × 10^8^	OR	5.99 ± 0.51^a^	11/11 (100)^a^	1/11 (9.09)^d^	5/11 (45.45)^cd^
6		IT	5.11 ± 0.47^a^	12/12 (100)^a^	11/12 (91.66)^a^	12/12 (100)^a^

^a–d^Different superscripts within columns indicate significant differences *P* < 0.05, n = 12/group.

^1^Chicks were challenged with *Salmonella* Enteritidis on d 7 intratracheally (IT) or orally (OR) at concentrations of 1.5 × 10^4^ or 2 × 10^6^ or 1 × 10^8^ cfu/chick. Twenty-four hours postchallenge, ceca-cecal tonsils were cultured to enumerate log_10_ *Salmonella* Enteritidis per gram of ceca content and the data were expressed as mean ± SEM. Ceca-cecal tonsils, trachea, and liver and spleen enrichment data were expressed as positive/total chickens for each tissue sampled (% in parentheses).

Historically, we have experienced difficulty consistently infecting older chickens with low doses of *Salmonella* in our laboratory. Although not proven by these experiments, the possibility that respiratory transmission is more effective might explain the relative difficulty in consistently infecting chicks via oral administration under laboratory conditions, as opposed to the apparent ease of transmission under commercial conditions, is an intriguing concept. The ability of *Salmonella* to infect chickens at lower doses (as low as 100 cells) via the respiratory route needs to be further investigated; nevertheless, this could support the studies (discussed above) establishing the relationship between the particles, size and number of particles relative to the concentration of *Salmonella* generated in the air. These studies estimated the concentration of airborne *Salmonella* to be up to 3.3 × 10^2^ to 1.2 × 10^4^ cfu m^3^ of air (Wathes et al., 1988; Baskerville et al., 1992; Lever and Williams, 1996; McDermid and Lever, 1996; Fallschissel et al., 2009). Thus, it is conceivable that under field conditions airborne *Salmonella* concentrations are able to infect poultry by this route of infection. The minimum infectious dose of *Salmonella* to infect chickens via the respiratory route in our study was similar to that found to produce disease in sentinel mice (150 cfu of *Salmonella* Typhimurium; Wathes et al., 1988).

Furthermore, previous studies to date have not described the subsequent fate of *Salmonella* infecting the respiratory system, while evaluating the infection via the respiratory route (Stearns et al., 1987; Wathes et al., 1988; Nganpiep and Maina, 2002; Harbaugh et al., 2006; Oliveira et al., 2006). In this regard, both the cecal cfu recovery data and LS incidence data from the present experiments provided evidence that *Salmonella* administered IT could colonize ceca, potentially involving a systemic route, as suggested by the increased recovery from LS samples (Table 1). In experiment 2, trial 1, 33% of LS samples were positive for *Salmonella* Enteritidis given IT compared with 0% given OR (Table 2). The incidence data for trial 2 displayed a similar trend as the preliminary trials. Significantly higher positive LS were observed at all 3 IT challenged doses, with 6/12 (50%), 10/12 (83.33%), and 11/12 (91.66%) positive LS samples compared with OR challenged groups with 0/12 (0%), 2/12 (16.66%), and 1/11 (9.09%) positive samples, for respective doses, all together indicating systemic organ invasion of *Salmonella* post-IT challenge (Table 3). Taken together, the results from this study may suggest that systemic infection, leading to biliary clearance, was responsible for the intestinal infections from the IT-challenged chicks, although other mechanisms are possible.

One mode by which *Salmonella* could infect and systemically spread is by entering the blood directly and causing bacteremia. This mode is quite conceivable considering previous studies proposing a disrupted epithelial barrier of the developing bronchus-associated lymphoid tissue structure, endocytosis by epithelial cells and further transport to the interstitial matrix, and the high prevalence of blood gas barrier breaks and epithelial epithelial cell connections (West and Mathieu-Costello, 1995, 1999; Nganpiep and Maina, 2002; Kiama et al., 2008). Alternatively, various reports have described the presence of an efficient phagocytic system in chickens, involving phagocytic epithelial lining, interstitial macrophages in the blood gas barrier, and infiltrating free avian respiratory macrophages (Karnovsky and Lazdins, 1978; Mensah and Brain, 1982; Nagaraja et al., 1984; Curtiss and Kelly, 1987; Toth et al., 1992; Maina and Cowley, 1998; Nganpiep and Maina, 2002; Kiama et al., 2008). *Salmonella*, having an established intracellular lifestyle and known to systemically disseminate via macrophages when given orally (Vazquez-Torres et al., 2000; Okamura et al., 2005), could potentially infect chickens via a similar pathway following respiratory exposure.

Further, there have been speculations that *Salmonella* infecting via the respiratory route might acquire increased virulence traits and hence infect chickens efficiently at low doses compared with the oral route. For example, Leach et al. (1999) observed a marked increase in reported cases of human salmonellosis caused by a multi-antibiotic-resistant strain of *Salmonella* Typhimurium definitive type 104 in England and Wales over the period of 1995 to 1998. They hypothesized and successfully tested the infection via the aerosol route and were able to reproduce the desired infection, wherein the frequency of *Salmonella* isolation from eggs rose from 2.1% following oral challenge to 14 to 25%, and the frequency of isolation from muscle rose from 0 to 27% following aerosol infection. Based on their observations, they suggested a greater virulence of the pathogen when given by aerosol. Even though this may suggest increased virulence, it is entirely possible that higher rates of egg and muscle tissue contamination have more to do with the respiratory route being a much simpler and more vulnerable route for *Salmonella* entry rather than necessarily involving virulence. Experiment 3, employing a relatively less enteroinvasive *Salmonella* Typhimurium as the challenge pathogen, provided support in this regard. Although comparing between experiments, cecal and LS incidence at all doses given IT were numerically lower for *Salmonella* Typhimurium compared with *Salmonella* Enteritidis. Nevertheless, IT administration was equally effective for infecting the CCT of chickens compared with OR, at all challenge doses (Table 4).

**Table 4.** Evaluation of intratracheal infection of chickens with *Salmonella* Typhimurium (experiment 3)^1^

Group	Dose, cfu of *Salmonella* Typhimurium/chick	Route of challenge	Ceca-cecal tonsil	Liver and spleen	Trachea
1	1.5 × 10^4^	OR	4/12 (33.33)^b^	0/12 (0)^b^	0/12 (0)^c^
2		IT	3/12 (25)^b^	1/12 (8.33)^b^	8/12 (66.66)^b^
3	2.5 × 10^6^	OR	11/12 (91.66)^a^	0/12 (0)^b^	0/12 (0)^c^
4		IT	9/12 (75)^a^	1/12 (8.33)^b^	12/12 (100)^a^
5	1 × 10^8^	OR	12/12 (100)^a^	2/12 (16.66)^b^	2/12 (16.66)^c^
6		IT	10/12 (83.33)^a^	9/12 (75)^a^	12/12 (100)^a^

^a–c^Different superscripts within columns indicate significant differences *P* < 0.05, n = 12/group.

^1^Chicks were challenged with *Salmonella* Typhimurium on d 7 intratracheally (IT) or orally (OR) at concentrations of 1.5 × 10^4^ or 2.5 × 10^6^ or 1 × 10^8^ cfu/chick. Twenty-four hours postchallenge, chicks were humanely killed, and ceca-cecal tonsils, trachea, and liver and spleen were cultured in an enrichment broth. The enrichment data were expressed as positive/total chickens for each tissue sampled (% in parentheses).

Overall, our data suggest that the respiratory route might be a viable portal of entry for *Salmonella* in poultry. Ongoing studies are aimed at further evaluation of this hypothesis using bioaerosol administration. Clarification of the potential importance of the respiratory tract for *Salmonella* transmission under field conditions may be of critical importance as efforts to develop intervention strategies to reduce transmission of these pathogens in poultry.

## References

[r1] Baskerville A., Humphrey T., Fitzgeorge R., Cook R., Chart H., Rowe B., Whitehead A. (1992). Airborne infection of laying hens with *Salmonella enteritidis* phage type 4.. Vet. Rec..

[r2] Baykov B., Stoyanov M. (1999). Microbial air pollution caused by intensive broiler chicken breeding.. FEMS Microbiol. Ecol..

[r3] Berrang M., Cox N., Bailey J. (1995). Measuring air-borne microbial contamination of broiler hatching cabinets.. J. Appl. Poult. Res..

[r4] Blaser M. J., Newman L. S. (1982). A review of human salmonellosis: I. Infective dose.. Rev. Infect. Dis..

[r5] Brooks J., McLaughlin M., Scheffler B., Miles D. (2010). Microbial and antibiotic resistant constituents associated with biological aerosols and poultry litter within a commercial poultry house.. Sci. Total Environ..

[r6] Bull S., Allen V., Domingue G., Jørgensen F., Frost J., Ure R., Whyte R., Tinker D., Corry J., Gillard-King J. (2006). Sources of *Campylobacter* spp. colonizing housed broiler flocks during rearing.. Appl. Environ. Microbiol..

[r7] Byrd J., DeLoach J., Corrier D., Nisbet D., Stanker L. (1999). Evaluation of *Salmonella* serotype distributions from commercial broiler hatcheries and grower houses.. Avian Dis..

[r8] Chinivasagam H., Tran T., Maddock L., Gale A., Blackall P. (2009). Mechanically ventilated broiler sheds: A possible source of aerosolized *Salmonella*, *Campylobacter*, and *Escherichia coli*.. Appl. Environ. Microbiol..

[r9] Clouser C., Doores S., Mast M., Knabel S. (1995). The role of defeathering in the contamination of turkey skin by *Salmonella* species and *Listeria monocytogenes*.. Poult. Sci..

[r10] Corrier D., Byrd J., Hargis B., Hume M., Bailey R., Stanker L. (1999). Presence of *Salmonella* in the crop and ceca of broiler chickens before and after preslaughter feed withdrawal.. Poult. Sci..

[r11] Cox J., Pavic A. (2010). Advances in enteropathogen control in poultry production.. J. Appl. Microbiol..

[r12] Curtiss R., Kelly S. M. (1987). *Salmonella*
*typhimurium* deletion mutants lacking adenylate cyclase and cyclic AMP receptor protein are avirulent and immunogenic.. Infect. Immun..

[r13] Davies R., Wray C. (1994). An approach to reduction of *Salmonella* infection in broiler chicken flocks through intensive sampling and identification of cross-contamination hazards in commercial hatcheries.. Int. J. Food Microbiol..

[r14] Dibner J., Knight C., Kitchell M., Atwell C., Downs A., Ivey F. (1998). Early feeding and development of the immune system in neonatal poultry.. J. Appl. Poult. Res..

[r15] Douwes J., Thorne P., Pearce N., Heederik D. (2003). Bioaerosol health effects and exposure assessment: Progress and prospects.. Ann. Occup. Hyg..

[r16] Dungan R. S. (2010). Board-Invited Review: Fate and transport of bioaerosols associated with livestock operations and manures.. J. Anim. Sci..

[r17] Fallschissel K., Kämpfer P., Jäckel U. (2009). Direct detection of *Salmonella* cells in the air of livestock stables by real-time PCR.. Ann. Occup. Hyg..

[r18] Fallschissel K., Klug K., Kämpfer P., Jäckel U. (2010). Detection of airborne bacteria in a German turkey house by cultivation-based and molecular methods.. Ann. Occup. Hyg..

[r19] Galanis E., Wong D. M. L. F., Patrick M. E., Binsztein N., Cieslik A., Chalermchaikit T., Aidara-Kane A., Ellis A., Angulo F. J., Wegener H. C. (2006). World Health Organization Global Salm-Surv. Web-based surveillance and global *Salmonella* distribution, 2000–2002.

[r20] Gast R. K., Mitchell B. W., Holt P. S. (2004). Detection of airborne *Salmonella enteritidis* in the environment of experimentally infected laying hens by an electrostatic sampling device.. Avian Dis..

[r21] Halevy O., Geyra A., Barak M., Uni Z., Sklan D. (2000). Early posthatch starvation decreases satellite cell proliferation and skeletal muscle growth in chicks.. J. Nutr..

[r22] Harbaugh E., Trampel D., Wesley I., Hoff S., Griffith R., Hurd H. (2006). Rapid aerosol transmission of *Salmonella* among turkeys in a simulated holding-shed environment.. Poult. Sci..

[r23] Heber A. J., Peugh M. W., Lutgring K. R., Zimmerman N. J., Linton R. H. (2006). Poultry slaughtering plants: Concentrations of microbial aerosols in poultry slaughtering and processing plants.. ASHRAE Transact..

[r24] Henderson S., Vicente J., Pixley C., Hargis B., Tellez G. (2008). Effect of an early nutritional supplement on broiler performance.. Int. J. Poult. Sci..

[r25] Hooshmand M. (2006). Effect of early feeding programs on broiler performance.. Int. J. Poult. Sci..

[r26] Jarquin R., Hanning I., Ahn S., Ricke S. C. (2009). Development of rapid detection and genetic characterization of *Salmonella* in poultry breeder feeds.. Sensors (Basel).

[r27] Juul-Madsen H. R., Su G., Sørensen P. (2004). Influence of early or late start of first feeding on growth and immune phenotype of broilers.. Br. Poult. Sci..

[r28] Karnovsky M. L., Lazdins J. K. (1978). Biochemical criteria for activated macrophages.. J. Immunol..

[r29] Kiama S., Adekunle J., Maina J. (2008). Comparative in vitro study of interactions between particles and respiratory surface macrophages, erythrocytes, and epithelial cells of the chicken and the rat.. J. Anat..

[r30] Leach S. A., Williams A., Davies A. C., Wilson J., Marsh P. D., Humphrey T. J. (1999). Aerosol route enhances the contamination of intact eggs and muscle of experimentally infected laying hens by *Salmonella*
*typhimurium* DT104.. FEMS Microbiol. Lett..

[r31] Lever M., Williams A. (1996). Cross-infection of chicks by airborne transmission of *Salmonella enteritidis* PT4.. Lett. Appl. Microbiol..

[r32] López F. E., de las Mercedes Pescaretti M., Morero R., Delgado M. A. (2012). *Salmonella* Typhimurium general virulence factors: A battle of David against Goliath?. Food Res. Int..

[r33] Maina J. N., Cowley H. M. (1998). Ultrastructural characterization of the pulmonary cellular defences in the lung of a bird, the rock dove, *Columba livia*.. Proc. Biol. Sci..

[r34] McCapes R., Osburn B., Riemann H. (1991). Safety of foods of animal origin: Model for elimination of *Salmonella* contamination of turkey meat.. J. Am. Vet. Med. Assoc..

[r35] McDermid A., Lever M. (1996). Survival of *Salmonella enteritidis* PT4 and *Salm. typhimurium* Swindon in aerosols.. Lett. Appl. Microbiol..

[r36] Mensah G. A., Brain J. D. (1982). Deposition and clearance of inhaled aerosol in the respiratory tract of chickens.. J. Appl. Physiol..

[r37] Mitchell B., Buhr R., Berrang M., Bailey J., Cox N. (2002). Reducing airborne pathogens, dust and *Salmonella* transmission in experimental hatching cabinets using an electrostatic space charge system.. Poult. Sci..

[r38] Nagaraja K. V., Emery D. A., Jordan K. A., Sivanandan V., Newman J. A., Pomeroy B. S. (1984). Effect of ammonia on the quantitative clearance of *Escherichia coli* from lungs, air sacs, and livers of turkeys aerosol vaccinated against *Escherichia coli*.. Am. J. Vet. Res..

[r39] NRC (1994). Nutrient Requirements of Poultry.

[r40] Nganpiep L., Maina J. (2002). Composite cellular defence stratagem in the avian respiratory system: Functional morphology of the free (surface) macrophages and specialized pulmonary epithelia.. J. Anat..

[r41] Noy Y., Geyra A., Sklan D. (2001). The effect of early feeding on growth and small intestinal development in the posthatch poult.. Poult. Sci..

[r42] Noy Y., Sklan D. (1998). Metabolic responses to early nutrition.. J. Appl. Poult. Res..

[r43] Okamura M., Lillehoj H. S., Raybourne R. B., Babu U. S., Heckert R. A., Tani H., Sasai K., Baba E., Lillehoj E. P. (2005). Differential responses of macrophages to *Salmonella enterica* serovars Enteritidis and Typhimurium.. Vet. Immunol. Immunopathol..

[r44] Oliveira C., Carvalho L., Garcia T. (2006). Experimental airborne transmission of *Salmonella* Agona and *Salmonella* Typhimurium in weaned pigs.. Epidemiol. Infect..

[r45] Ramirez G., Sarlin L., Caldwell D., Yezak C., Hume M., Corrier D., Hargis B. (1997). Effect of feed withdrawal on the incidence of *Salmonella* in the crops and ceca of market age broiler chickens.. Poult. Sci..

[r46] SAS Institute (2002). SAS User Guide. Version 9.1.

[r47] Sklan D., Noy Y. (2000). Hydrolysis and absorption in the small intestines of posthatch chicks.. Poult. Sci..

[r48] Stearns R. C., Barnas G., Walski M., Brain J. (1987). Deposition and phagocytosis of inhaled particles in the gas exchange region of the duck, *Anas platyrhynchos*.. Respir. Physiol..

[r49] Toth T. E., Curtiss R., Veit H., Pyle R., Siegel P. (1992). Reaction of the avian respiratory system to intratracheally administered avirulent *Salmonella typhimurium*.. Avian Dis..

[r50] Uni Z., Smirnov A., Sklan D. (2003). Pre-and posthatch development of goblet cells in the broiler small intestine: Effect of delayed access to feed.. Poult. Sci..

[r51] Vazquez-Torres A., Xu Y., Jones-Carson J., Holden D. W., Lucia S. M., Dinauer M. C., Mastroeni P., Fang F. C. (2000). *Salmonella* pathogenicity island 2-dependent evasion of the phagocyte NADPH oxidase.. Science.

[r52] Wathes C., Zaidan W., Pearson G., Hinton M., Todd N. (1988). Aerosol infection of calves and mice with *Salmonella typhimurium*.. Vet. Rec..

[r53] West J., Mathieu-Costello O. (1999). Structure, strength, failure, and remodeling of the pulmonary blood-gas barrier.. Annu. Rev. Physiol..

[r54] West J. B., Mathieu-Costello O. (1995). Stress failure of pulmonary capillaries as a limiting factor for maximal exercise.. Eur. J. Appl. Physiol. Occup. Physiol..

[r55] White P., Baker A., James W. (1997). Strategies to control *Salmonella* and *Campylobacter* in raw poultry products.. Rev. Sci. Tech..

[r56] Yi G., Allee G., Knight C., Dibner J. (2005). Impact of glutamine and oasis hatchling supplement on growth performance, small intestinal morphology, and immune response of broilers vaccinated and challenged with *Eimeria maxima*.. Poult. Sci..

[r57] Zar J. (1984). Biostatistical Analysis. Prentice Hall.

